# Producer–retailer integrated EMQ system with machine breakdown, rework failures, and a discontinuous inventory issuing policy

**DOI:** 10.1186/s40064-016-1980-4

**Published:** 2016-03-15

**Authors:** Singa Wang Chiu, Shin-Wei Chen, Yuan-Shyi Peter Chiu, Ting-Wei Li

**Affiliations:** Department of Business Administration, Chaoyang University of Technology, Taichung, 413 Taiwan; Department of Industrial Engineering and Management, Chaoyang University of Technology, Taichung, 413 Taiwan

**Keywords:** Manufacturing, Producer–retailer integrated system, Economic manufacturing quantity, Breakdown, Discontinuous issuing policy, Rework failures, Optimization

## Abstract

This study develops two extended economic manufacturing quantity (EMQ)-based models with a discontinuous product issuing policy, random machine breakdown, and rework failures. Various real conditions in production processes, end-product delivery, and intra-supply chains such as a producer–retailer integrated scheme are examined. The first model incorporates a discontinuous multi-delivery policy into a prior work (Chiu et al. in Proc Inst Mech Eng B J Eng 223:183–194, 2009) in lieu of their continuous policy. Such an enhanced model can address situations in supply chain environments, where finished products are transported to outside retail stores (or customers). The second model further combines retailer’s stock holding costs into the first model. This extended EMQ model is applicable in situations in present-day manufacturing firms where finished products are distributed to company’s own retail stores (or regional sales offices) and stocked there for sale. Two aforementioned extended EMQ models are investigated, respectively. Mathematical modeling along with iterative algorithms are employed to derive the optimal production run times that minimize the expected total system costs, including the costs incurred in production units, transportation, and retail stores, for these integrated EMQ systems. Numerical examples are provided to demonstrate the practical application of the research results.

## Background

This study explores the optimal production run time for a producer–retailer integrated economic manufacturing quantity (EMQ) model with rework failures, random machine breakdown, and a discontinuous inventory issuing policy. The EMQ model employed mathematical techniques to balance the production setup cost and inventory holding cost in a production cycle to derive the most economic manufacturing quantity that minimizes the long-run average production–inventory costs per unit time (Taft 1918; Wagner and Whitin 1958; Nahmias 2009). The classic EMQ model implicitly assumes that production equipment is in perfect condition and all items produced are of perfect quality. However, in real manufacturing environments, due to process deterioration or other uncontrollable factors, both production of items of imperfect quality and machine breakdown are inevitable. Unsurprisingly, many studies have been carried out to enhance the classic EMQ model by addressing issues of imperfect product quality and random machine breakdowns (Barlow and Proschan 1965; Shih 1980; Bielecki and Kumar 1988; Grosfeld-Nir and Gerchak 2002; Inderfurth et al. 2006; Hishamuddin et al. 2014; Chiu and Chang 2014; Lin et al. 2014; Wu et al. 2014; Khedlekar et al. 2014; Pal et al. 2015; Ocampo 2015; Chiu et al. 2015a, b, c). Henig and Gerchak (1990) conducted a comprehensive analysis of a general periodic review production/inventory model with variable yield. Groenevelt et al. (1992) proposed two production control policies to cope with machine breakdown. The first policy considers that the production of an interrupted lot will not be resumed after a breakdown (i.e., the no resumption or NR policy), while the second assumes that the production of an interrupted lot resumed immediately after production is restored and if the current on-hand inventory is below a certain threshold level (i.e., the abort-resume or AR policy). Wee (1993) proposed an economic production policy for deteriorating items with partial back-ordering. Two numerical examples were used to illustrate this proposed theory, and the computational results indicated that the policy led to a lower cost. Gopalan and Kannan (1994) examined a two-stage transfer-line production system with inspection and rework. Transient state characteristics were analyzed for the system, subject to an initial buffer of infinite capacity, inspection at both inter- and end-stages, and rework. A stochastic model was developed to study the system, and as a result, some explicit analytical expressions of system characteristics were revealed. Moinzadeh and Aggarwal (1997) investigated a production–inventory system with random disruptions. They proposed an (s, S) policy for the system in which the time between breakdowns is exponential, restoration times are constant, and excess demand is backordered. A procedure for deriving optimal values from the policy was developed, and the system parameters that minimize the expected total cost per unit time were examined. Jabal Ameli et al. (2008) proposed a multi-objective integer linear programming approach for cell formation problems with alternative process routings and machine reliability issues. Their study aimed to simultaneously minimize total cost and maximize system reliability. Unlike the traditional reliability evaluation approaches, the approach used in their study was to model machine unreliability in terms of cost and time-based effects. Using the e-constraint method as an optimization tool for multi-objective programming, a numerical example was provided to demonstrate the capability of the proposed model to evaluate various effects of reliability issues. Chiu et al. (2009) studied the optimal production run time in an EMQ model with imperfect rework and Poisson machine breakdowns under the abort/resume (A/R) control policy. In their proposed system, a random defective rate is assumed and all defective items are reworked at the end of regular production, and there exists a certain percentage of rework failures. The system is subject to random breakdowns and the A/R inventory control policy is adopted when breakdowns occur. Mathematical modeling was used, and theorems related to conditional convexity and bounds of optimal production run times were proposed and proved in their study. A recursive searching algorithm was developed to locate the optimal run time that minimizes the expected production–inventory costs.

Unlike the implicit assumption of *continuous inventory issuing policy* in the classic EMQ model, in real-life supply chain systems, the most commonly adopted policy for distribution of finished products is the discontinuous (periodic) multiple delivery policy. Schwarz (1973) studied a deterministic one-warehouse N-retailer inventory problem with a continuous review policy. Schwarz’s study aimed to determine the stocking policy that minimizes the average system cost. Goyal and Gupta (1989) reviewed and classified the vendor–buyer coordinated inventory models. They not only provided a scheme for models classification but also identified a few possible directions for future research. Sarker and Parija (1994) derived the optimal batch size for a production system operating under a fixed-quantity and periodic delivery policy. They studied a manufacturing system where raw materials were procured from vendors, and then processed and converted into finished items. An integrated inventory model was proposed and analyzed to simultaneously determine the optimal ordering policy for raw materials and the most economical production batch size so that total system costs could be minimized. Hill (1999) determined the optimal production and shipment policy for the single-vendor single-buyer integrated production–inventory problem. It was assumed that a vendor manufactures a product in batches at a finite rate and ships it to a customer who then consumes the product at a fixed rate. The objective was to determine a purchasing and production schedule that minimizes the overall system costs. As a result, a global optimal solution was derived. Viswanathan and Piplani (2001) proposed a model with a specific vendor–buyer coordinating discipline to analyze the benefit of supply chain inventories by using common replenishment epochs or time periods. A one-vendor, multi-buyer supply chain for a single product was analyzed under a specific strategy, whereby the vendor specifies common replenishment periods and requires all buyers to replenish only at those time periods. In return, the vendor offers a price discount to convince buyers to accept this strategy. As a result, they determined the optimal replenishment period and the price discount to be offered by the vendor for the proposed model. Giri and Maiti (2012) studied a supply chain model for a deteriorating product with time-varying demand and production rate. A single-vendor single-buyer two-echelon supply chain model was examined, whereby the buyer sells a seasonal product and its inventory is subject to deterioration, and the vendor’s production rate is dependent on the buyer’s demand rate, which is a linear function of time. Further, some nonconforming items may be randomly produced during a production run. Mathematical modeling was used to derive the average cost function of the proposed supply chain system. An algorithm for finding the optimal solution was developed. Additional studies (Kreng and Chen 2007; Gong and Chen 2012; Glock 2012; Wee and Widyadana 2013; Chiu et al. 2014; Glock et al. 2014; Tseng et al. 2014; Safaei 2014; Rodger 2014) focused on various aspects of supply chain optimization.

To address real-life production–shipment situations, this study first extends Chiu et al.’s work (2009) by incorporating a discontinuous multi-delivery policy into their model in lieu of a continuous policy assumption to examine situations in real supply chain environments, where finished products are transported to retail stores (or customers) outside the production units. Next, the retailer’s stock holding cost is incorporated into the proposed model in the second part of the study to explicitly deal with situations in present-day manufacturing firms where finished products are distributed to companies’ own retail stores (or regional sales offices) and stocked there for sale. Accordingly, two separate extended EMQ models are developed, and with the aid of mathematical modeling and optimization techniques, we derive the optimal production run times that minimize the expected total system costs, including the costs incurred in production units, transportation, and retail stores, for these integrated EMQ systems. This study intends to fill the research gap in this specific area.

## Model 1: Description, modeling, and solution process

The first proposed model in this study explores the optimal production run time for an economic manufacturing quantity model with a discontinuous inventory issuing policy, random machine breakdown, and rework failures. The first model extends the work of Chiu et al. (2009) by incorporating a discontinuous multi-delivery policy into their model in lieu of a continuous policy assumption. This enhanced model can be used to address the situations in real supply chain environments, where finished products are transported to the retail store or customer outside the production units.

Consider that a manufacturing system can produce a product at an annual production rate *P*_1_ and the demand rate for this product is *λ* units per year. The production process may randomly produce *x* portion of defective items at a rate *d*_1_, where *d*_1_ = *P*_1_*x*. In order to sustain regular operations (i.e., avoid the occurrence of shortage) (*P*_1_ − *d*_1_ − *λ*) > 0 must be satisfied. All products manufactured are screened and the unit inspection cost is included in the unit production cost *C*. All nonconforming items are reworked at a rate of *P*_2_, and the rework process starts immediately after the completion of the regular process. Since 100 % of reworks are not successful, a *θ*_1_ portion (where 0 ≤ *θ*_1_ < 1) of reworked items fails and becomes scraps.

**Fig. 1:**
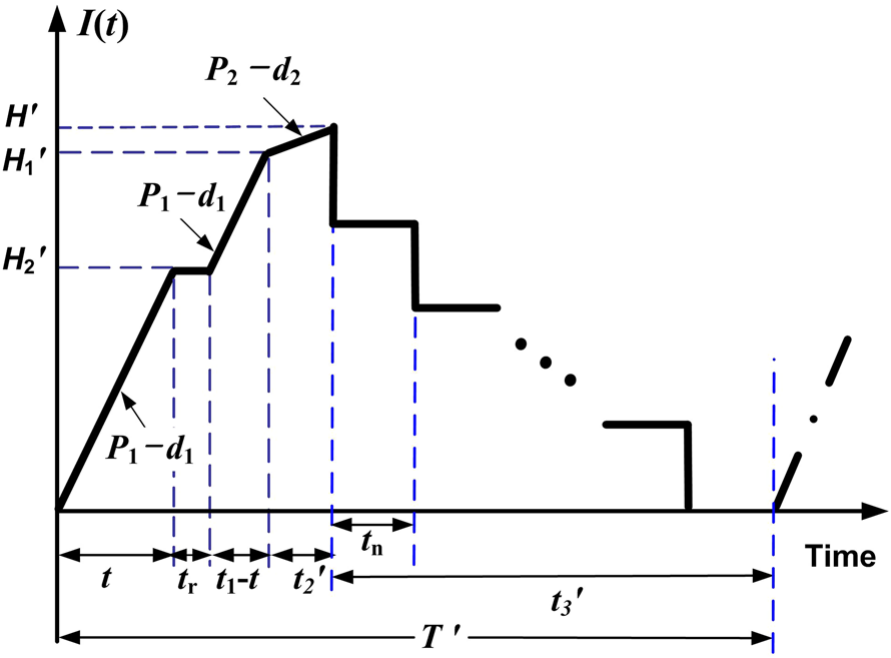
On-hand inventory level of perfect quality products in the extended EMQ model 1 with rework failures, machine breakdown, and multi-delivery policy

It is assumed that finished products can only be delivered to the retail store if the entire production lot is quality assured after rework. A multi-delivery policy is used for transporting finished items. Under such a discontinuous product issuing policy, *n* fixed quantity installments of the finished lot are delivered at a fixed interval of time during delivery time $t_{3}^{'}$ (see Fig. 1). Furthermore, during production time the machine is subject to a random breakdown which follows a Poisson distribution. The abort/resume (A/R) inventory control policy is used when a breakdown takes place. Under such a policy, the machine goes under repair immediately after a breakdown occurs, a constant machine repair time is assumed, and the interrupted lot will be resumed right after the restoration of machine (see Fig. 1).

In addition to the unit production cost *C*, other cost-related parameters used in the proposed modeling and analysis include the production setup cost *K*, a fixed cost *M* for machine repairing, unit holding cost *h* at the producer’s end, unit rework cost *C*_R_, holding cost *h*_1_ for each reworked item, disposal cost per scrap item *C*_S_, fixed delivery cost *K*_1_ per shipment, unit shipping cost *C*_T_, and unit holding cost *h*_2_ at the retailer’s end. Additional notation used is listed below:

*t*_1_: the production time (i.e. uptime) to be determined in the proposed EMQ model,
*t*: production time before a random breakdown occurs,
*β*: number of breakdowns per year, a random variable which follows Poisson distribution,
*t*_r_: machine repair time,
$$t_{2}^{'}$$:
time needed to rework the defective items (in the case of breakdown occurrence),
$$t_{3}^{'}$$:
the time needed to transport all available perfect quality items (in the case of breakdown occurrence),
$$H_{2}^{'}$$:
the level of on-hand inventory in units when random breakdown takes place,
$$H_{1}^{'}$$:
the level of on-hand inventory in units when regular production ends (in the case of breakdown occurrence),
$$H^{'}$$:
the maximum level of on-hand inventory in units when regular production ends (in the case of breakdown occurrence),
*I*(*t*): level of on-hand perfect quality items at time *t*,
*I*_d_(*t*): level of on-hand defective items at time *t*,
*I*_S_(*t*): level of on-hand scrap items at time *t*,
*Q*: production lot size per cycle,
$$T^{'}$$:
cycle length (in the case of breakdown occurrence),
*h*_3_: unit holding cost for safety stock,
*TC*_1_(*t*_1_): the total production–inventory–delivery costs per cycle (in the case of breakdown occurrence),
*E*[*TC*_1_(*t*_1_)]: the expected production–inventory–delivery costs per cycle (in the case of breakdown occurrence),
*t*_2_: time required to rework the defective items (in the case of no breakdown occurrence),
*t*_3_: time required for transport all available perfect quality items (in the case of no breakdown occurrence),
*H*_1_: the level of on-hand inventory in units when regular production ends (in the case of no breakdown occurrence),
*H*: the maximum level of on-hand inventory in units when the rework process ends (in the case of no breakdown occurrence),
*TC*_2_(*t*_1_): total production–inventory–delivery costs per cycle when no breakdown occurrence,
*E*[*TC*_2_(*t*_1_)]: the expected production–inventory–delivery costs per cycle (in the case of no breakdown occurrence),
*T*: cycle length (in the case of no breakdown occurrence),
*TCU*(*t*_1_): the total production–inventory–delivery costs per unit time whether a breakdown takes place or not,
*E*[*TCU*(*t*_1_)]: the long-run expected production–inventory–delivery costs per unit time whether or not a breakdown takes place,
***T***: the cycle length whether or not a machine breakdown takes place.

Suppose *t* denotes time before a random breakdown taking place during production uptime *t*_1_, then the following two different cases must be analyzed, respectively.

### Case 1: A breakdown occurs during uptime *t*_1_

**Fig. 2:**
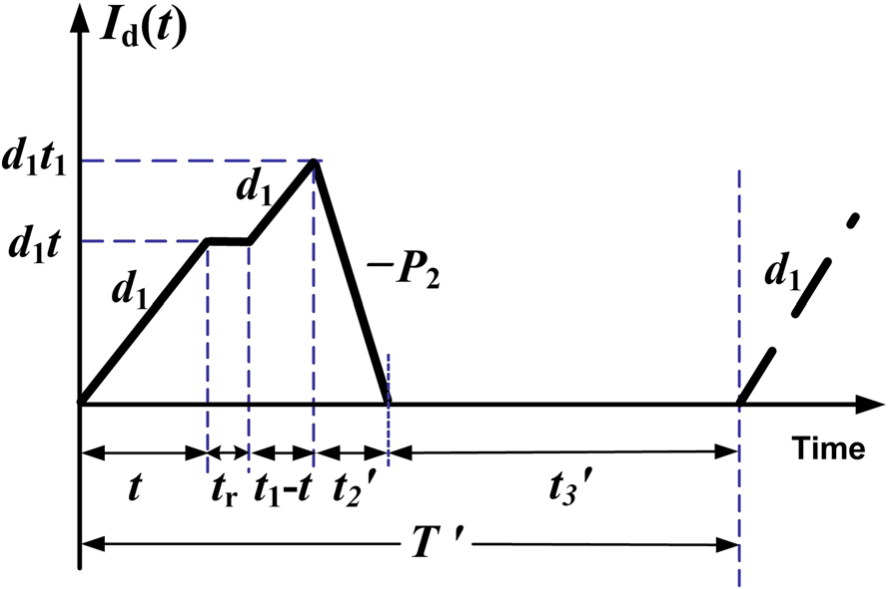
On-hand inventory level of defective products in the extended EMQ model 1

In this case, *t* < *t*_1_. That is the time before a random breakdown taking place is shorter than the production uptime. In other words, a machine breakdown takes place during the production process. Under the AR inventory policy, the machine goes under repair immediately, and, once it is fixed and restored, the production of interrupted lot is resumed right away. The on-hand inventory level of perfect quality products at the time a breakdown occurs is $H_{2}^{'}$ (refer to Fig. 1), and the level of on-hand inventory remains at $H_{2}^{'}$ until the machine is repaired. While $H_{1}^{'}$ denotes the level of on-hand inventory in units when regular production of the remaining of interrupted lot is completed. Then, the reworking of defective products begins at a rate of *P*_2_ per year (see Fig. 2) and the maximum number of defective items per cycle is given in Eq. (1).

1 $$d_{1} t_{1} = xQ = xP_{1} t_{1}$$

**Fig. 3:**
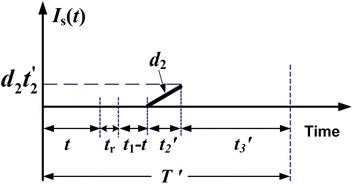
On-hand inventory level of scrap items in the extended EMQ model 1

It is assumed that 100 % of reworks are not successful, a *θ*_1_ portion of reworked items fails and becomes scrap during $t_{2}^{'}$ (see Fig. 3) and Eq. (2) shows maximum number of scrap items.

2 $$\theta_{1} xQ = d_{2} t_{2}^{'}$$

Upon the completion of rework, the on-hand inventory level perfect quality products is $H^{'}$ (Fig. 1). Next, in product delivery time $t_{3}^{'}$, fixed quantity of *n* installments of finished batch is delivered to the customer at a fixed interval of time. It can be seen from Fig. 1 that production cycle time $T^{'}$ is

3 $$T^{'} = t + t_{r} + (t_{1} - t) + t_{2}^{'} + t_{3}^{'}$$

In the case of breakdown occurrence, the total production–inventory–delivery costs per cycle, *TC*_1_(*t*_1_) consists of (1) the variable production cost; (2) production setup cost; (3) machine repair cost; (4) rework cost; (5) disposal cost for scraps; (6) fixed and the variable product transporting costs; (7) inventory holding cost during rework; (8) holding cost for safety stocks (i.e., stocks to prevent shortage occurrence due to a machine breakdown); and (9) inventory holding costs during the entire production cycle. Thus, *TC*_1_(*t*_1_) can be expressed as

4 $$\begin{aligned} TC_{1} (t_{1} ) & = C\left( {P_{1} t_{1} } \right) + K + M + C_{R} \left( {t_{1} P_{1} x} \right) + C_{S} \left( {t_{1} P_{1} x\theta_{1} } \right) + nK_{1} + C_{T} \left[ {t_{1} P_{1} (1 - \theta_{1} x)} \right] + h_{1} \frac{{d_{1} t_{1} }}{2}t_{2}^{'} + h_{3} (\lambda t_{r} )T^{'} \\ & \quad + h\left[ {\frac{{H_{1}^{'} + d_{1} t_{1} }}{2}t_{1} + (H_{2}^{'} + d_{1} t)t_{r} + \frac{{H_{1}^{'} + H^{'} }}{2}(t_{2}^{'} ) + \frac{n - 1}{2n}H^{'} t_{3}^{'} } \right] \\ \end{aligned}$$

In this study, it is assumed that *x* is a random variable with a known probability density function, thus the expected values of *x* are used in our analysis to take the randomness of *x* into account. By substituting all related system parameters into Eq. (4) (Chiu et al. 2009) and with further derivations, the expected system costs per cycle for the case of breakdown occurrence, E[*TC*_1_(*t*_1_)] can be obtained as

5 $$\begin{aligned} E[TC_{1} (t_{1} )] & = K + M + nK_{1} + htP_{1} g + \left[ \begin{aligned} CP_{1} + C_{R} P_{1} E[x] + C_{S} P_{1} \theta_{1} E[x] + C_{T} P_{1} (1 - \theta_{1} E[x]) \hfill \\ + h_{3} P_{1} g(1 - \theta_{1} E[x]) - \frac{{hP_{1} g(1 - \theta_{1} E[x])}}{2}\left( {1 - \frac{1}{n}} \right) \hfill \\ \end{aligned} \right] \cdot t_{1} \\ & + \left[ \begin{aligned} \frac{{hP_{1} \theta_{1} E[x]}}{2} + \frac{{hP_{1}^{2} E[x]}}{{P_{2} }}(1 - E[x]) + \frac{{hP_{1}^{2} }}{2\lambda }(1 - \theta_{1} E[x])^{2} \left( {1 - \frac{1}{n}} \right) \hfill \\ +\quad \frac{{hP_{1} }}{2n}(1 - \theta_{1} E[x]) + \frac{{hP_{1}^{2} E[x]}}{{2P_{2} n}}(1 - \theta_{1} E[x]) + \frac{{h_{1} P_{1}^{2} E[x]^{2} }}{{2P_{2} }} \hfill \\ \end{aligned} \right]t_{1}^{2} \\ \end{aligned}$$

### Case 2: No breakdown occurs during uptime t_1_

In this case, *t* > *t*_1_. That is the time before a random breakdown taking place is longer than the production uptime. In other words, no breakdown occurs during the production process (Chiu et al. 2009).

In case 2 the production cycle length is *T* = *t*_1_ + *t*_2_ + *t*_3_, and total production–inventory–delivery costs per cycle (in no breakdown occurrence case) *TC*_2_(*t*_1_) is

6 $$\begin{aligned} TC_{2} (t_{1} ) &= C\left( {P_{1} t_{1} } \right) + K + C_{R} \left( {xt_{1} P_{1} } \right) + C_{S} \left( {\theta_{1} xt_{1} P_{1} } \right) + nK_{1} + C_{T} \left[ {t_{1} P_{1} (1 - \theta_{1} x)} \right] \\ & \quad + h_{1} \frac{{d_{1} t_{1} }}{2}t_{2} + h_{3} (\lambda t_{r} )T + h\left[ {\frac{{H_{1} + d_{1} t_{1} }}{2}t_{1} + \frac{{H_{1} + H}}{2}(t_{2} ) + \frac{n - 1}{2n}Ht_{3} } \right] \\ \end{aligned}$$

To take randomness of defective items into account and substitute all related parameters in Eq. (6), and with further derivations E[*TC*_2_(*t*_1_)] can be obtained as follows:

7 $$\begin{aligned} E[TC_{2} (t_{1} )] & = K + nK_{1} + \left[ \begin{aligned} CP_{1} + C_{R} P_{1} E[x] + C_{S} \theta_{1} E[x]P_{1} \hfill \\ + C_{T} P_{1} (1 - \theta_{1} E[x]) + h_{3} P_{1} g(1 - \theta_{1} E[x]) \hfill \\ \end{aligned} \right]t_{1} \\ & \quad + \left[ \begin{aligned} \frac{{hP_{1} \theta_{1} E[x]}}{2} + \frac{{hP_{1}^{2} E[x]}}{{P_{2} }}\left( {1 - E[x]} \right) + \frac{{hP_{1}^{2} }}{2\lambda }\left( {1 - \theta_{1} E[x]} \right)^{2} \left( {1 - \frac{1}{n}} \right) \hfill \\ + \frac{{hP_{1} }}{2n}\left( {1 - \theta_{1} E[x]} \right) + \frac{{hP_{1}^{2} E[x]}}{{2P_{2} n}}\left( {1 - \theta_{1} E[x]} \right) + \frac{{h_{1} P_{1}^{2} E[x]^{2} }}{{2P_{2} }} \hfill \\ \end{aligned} \right]t_{1}^{2} \\ \end{aligned}$$

### Integration of the proposed EMQ models with and without breakdown

This study assumes a breakdown can occur randomly and it follows a Poisson distribution with mean equals to *β* per year. Let *f*(*t*) represent the probability density function of random time *t* before a breakdown occurs and *F*(*t*) denote the cumulative density function of *t*. Then, the expected production–inventory–delivery costs per unit time, E[*TCU*(*t*_1_)] is

8 $$E\left[ {TCU\left( {t_{1} } \right)} \right] = \frac{{\left\{ {\int_{ \, 0}^{{ \, t_{1} }} {E\left[ {TC_{1} \left( {t_{1} } \right)} \right]} f\left( t \right)dt + \int_{{ \, t_{1} }}^{ \, \infty } {E\left[ {TC_{2} \left( {t_{1} } \right)} \right]} f\left( t \right)dt} \right\}}}{{{\text{E}}[\varvec{T}]}}$$

The expected cycle length E[***T***] is

9 $${\text{E}}[\varvec{T}] = \int_{ \, 0}^{{ \, t_{1} }} { \, E\left[ {T^{'} } \right]} \, f\left( t \right)dt + \int_{{ \, t_{1} }}^{ \, \infty } { \, E\left[ T \right]} \, f\left( t \right)dt = \frac{{t_{1} P_{1} (1 - \theta_{1} E[x])}}{\lambda }$$

Since the number of breakdown per unit time is a random variable that follows a Poisson distribution with mean equal to *β*. The time between breakdowns obeys the Exponential distribution with density function *f*(*t*) = $\beta e^{ - \beta t}$, and the cumulative density function F(*t*) = $1 - e^{ - \beta t}$.

By substituting E[*TC*_1_(*t*_1_)], E[*TC*_2_(*t*_1_)], and E[***T***] into Eq. (8) and solving the integration of the mean time to breakdown in E[*TCU*(*t*_1_)], we obtain

10 $$\begin{aligned} E\left[ {TCU(t_{1} )} \right] = \frac{\lambda }{{\left( {1 - \theta_{1} E[x]} \right)}} \cdot \hfill \\ \quad \quad \left\{ \begin{aligned} \frac{{\left( {K + nK_{1} } \right)}}{{t_{1} P_{1} }} + \delta_{1} + \frac{{\delta_{2} t_{1} }}{2} + \left[ {\frac{M}{{P_{1} }} + \frac{hg}{\beta }} \right]\left( {\frac{{1 - e^{{ - \beta t_{1} }} }}{{t_{1} }}} \right) \hfill \\ - hg\left( {e^{{ - \beta t_{1} }} } \right) - \frac{{hg\left( {1 - \theta_{1} E[x]} \right)}}{2}\left( {1 - \frac{1}{n}} \right)\left( {1 - e^{{ - \beta t_{1} }} } \right) \hfill \\ \end{aligned} \right\} \hfill \\ \end{aligned}$$

where

11 $$\delta_{1} = \left[ {C + C_{R} E[x] + C_{S} \theta_{1} E[x] + C_{T} \left( {1 - \theta_{1} E[x]} \right) + h_{3} g\left( {1 - \theta_{1} E[x]} \right)} \right]$$

and

12 $$\delta_{2} = \left[ \begin{aligned} & \frac{{hP_{1} E[x]}}{{P_{2} }}\left( {1 - E[x]} \right) + \frac{{hP_{1} }}{\lambda }\left( {1 - \theta_{1} E[x]} \right)^{2} \left( {1 - \frac{1}{n}} \right) + h\theta_{1} E[x] \hfill \\ &+ \frac{h}{n}\left( {1 - \theta_{1} E[x]} \right) + \frac{{hP_{1} E[x]}}{{P_{2} n}}\left( {1 - \theta_{1} E[x]} \right) + \frac{{h_{1} P_{1} E[x]^{2} }}{{P_{2} }} \hfill \\ \end{aligned} \right]$$

### Determining the optimal production run time

With the aim of determining the optimal production run time $t_{1}^{*}$, we first must prove that E[*TCU*(*t*_1_)] is convex. Let *w*(*t*_1_) represent the following:

13 $$w\left( {t_{1} } \right) = \frac{{2\left( {K + nK_{1} } \right)\beta + 2\left( {1 - e^{{ - \beta t_{1} }} } \right)\delta_{4} }}{{\left[ {t_{1}^{2} P_{1} \beta^{2} \delta_{3} + \delta_{4} \left( {2 + \beta t_{1} } \right)} \right]\beta e^{{ - \beta t_{1} }} }}$$

#### **Theorem 1**

*E*[*TCU*(*t*_1_)] *is convex if 0* < *t*_1_ < *w*(*t*_1_).

Equation (14) shows the second derivative of E[*TCU*(*t*_1_)] with respect to *t*_1_.

14 $$\frac{{d^{2} E[TCU(t_{1} )]}}{{d^{2} t_{1}^{2} }} = \frac{\lambda }{{\left( {1 - \theta_{1} E[x]} \right)}} \cdot \left[ \begin{aligned} &\frac{{2\left( {K + nK_{1} } \right)}}{{t_{1}^{3} P_{1} }} - hg\left[ {1 - \frac{{\left( {1 - \theta_{1} E[x]} \right)}}{2}\left( {1 - \frac{1}{n}} \right)} \right]\left( {\beta^{2} e^{{ - \beta t_{1} }} } \right) \hfill \\ &+ \left[ {\frac{M}{{P_{1} }} + \frac{hg}{\beta }} \right]\left( {\frac{{2\left( {1 - e^{{ - \beta t_{1} }} } \right)}}{{t_{1}^{3} }} - \frac{{2\beta e^{{ - \beta t_{1} }} }}{{t_{1}^{2} }} - \frac{{\beta^{2} e^{{ - \beta t_{1} }} }}{{t_{1} }}} \right) \hfill \\ \end{aligned} \right]$$

The first term in the right-hand side (RHS) of Eq. (14) is positive because annual demand *λ* > 0. Therefore, we obtain

15 $${\text{if}}\left[ \begin{aligned} &\frac{{2\left( {K + nK_{1} } \right)}}{{t_{1}^{3} P_{1} }} - hg\left[ {1 - \frac{{\left( {1 - \theta_{1} E[x]} \right)}}{2}\left( {1 - \frac{1}{n}} \right)} \right]\left( {\beta^{2} e^{{ - \beta t_{1} }} } \right) \hfill \\ &+ \left[ {\frac{M}{{P_{1} }} + \frac{hg}{\beta }} \right]\left( {\frac{{2\left( {1 - e^{{ - \beta t_{1} }} } \right)}}{{t_{1}^{3} }} - \frac{{2\beta e^{{ - \beta t_{1} }} }}{{t_{1}^{2} }} - \frac{{\beta^{2} e^{{ - \beta t_{1} }} }}{{t_{1} }}} \right) \hfill \\ \end{aligned} \right] > 0\quad {\text{then}}\;\frac{{d^{2} E\left[ {TCU\left( {t_{1} } \right)} \right]}}{{dt_{1}^{2} }} > 0 \,$$

With further derivations, the left-hand size (LHS) in Eq. (15) becomes

16 $${\text{if}}\left[ \begin{aligned} &2\left( {K + nK_{1} } \right)\beta - t_{1}^{3} P_{1} \beta hg\left[ {1 - \frac{{\left( {1 - \theta_{1} E[x]} \right)}}{2}\left( {1 - \frac{1}{n}} \right)} \right]\left( {\beta^{2} e^{{ - \beta t_{1} }} } \right) \hfill \\ &+ \left( {M\beta + hgP_{1} } \right)\left[ {2\left( {1 - e^{{ - \beta t_{1} }} } \right) - 2t_{1} \beta e^{{ - \beta t_{1} }} - \beta^{2} t_{1}^{2} e^{{ - \beta t_{1} }} } \right] \hfill \\ \end{aligned} \right] > 0 \,$$

Let

17 $$\delta_{3} = hg\left[ {1 - \frac{{\left( {1 - \theta_{1} E[x]} \right)}}{2}\left( {1 - \frac{1}{n}} \right)} \right]\quad {\text{and}}\quad \delta_{4} = \left( {M\beta + hgP_{1} } \right) \,$$

then we have Eq. (16) as

18 $${\text{if}}\left[ \begin{aligned} & 2\left( {K + nK_{1} } \right)\beta - t_{1}^{3} P_{1} \beta \delta_{3} \left( {\beta^{2} e^{{ - \beta t_{1} }} } \right) \hfill \\ &+ \delta_{4} \left[ {2\left( {1 - e^{{ - \beta t_{1} }} } \right) - 2t_{1} \beta e^{{ - \beta t_{1} }} - \beta^{2} t_{1}^{2} e^{{ - \beta t_{1} }} } \right] \hfill \\ \end{aligned} \right] > 0 \,$$

Therefore, Eq. (15) becomes

19 $${\text{if }}\left[ \begin{aligned} &2\left( {K + nK_{1} } \right)\beta + 2\left( {1 - e^{{ - \beta t_{1} }} } \right)\delta_{4} \hfill \\ &- t_{1} \left[ {t_{1}^{2} P_{1} \beta^{2} \delta_{3} + \delta_{4} \left( {2 + \beta t_{1} } \right)} \right]\beta e^{{ - \beta t_{1} }} \hfill \\ \end{aligned} \right]{\text{ > 0}}\quad{\text{ then }}\frac{{d^{2} E\left[ {TCU\left( {t_{1} } \right)} \right]}}{{dt_{1}^{2} }}{ > 0 }$$

or

20 $$\therefore \frac{{d^{2} \, E\left[ {TCU\left( {t_{1} } \right)} \right]}}{{dt_{1}^{2} }} > 0\quad {\text{if}}\quad 0 < t_{1} < \frac{{2\left( {K + nK_{1} } \right)\beta + 2\left( {1 - e^{{ - \beta t_{1} }} } \right)\delta_{4} }}{{\left[ {t_{1}^{2} P_{1} \beta^{2} \delta_{3} + \delta_{4} \left( {2 + \beta t_{1} } \right)} \right]\beta e^{{ - \beta t_{1} }} }} = w\left( {t_{1} } \right)$$

If *E*[*TCU*(*t*_1_)*]* is convex, then to search for the optimal production run time $t_{1}^{*}$, we can set the first derivative of E[*TCU*(*t*_1_)] = 0 as follows:

21 $$\frac{{dE[TCU(t_{1} )]}}{{dt_{1} }} = \frac{\lambda }{{\left( {1 - \theta_{1} E[x]} \right)}} \cdot \left\{ \begin{aligned} &\frac{{ - \left( {K + nK_{1} } \right)}}{{t_{1}^{2} P}} + \frac{{\delta_{2} }}{2} + \delta_{3} \left( {\beta e^{{ - \beta t_{1} }} } \right) \hfill \\ &+ \left[ {\frac{M}{{P_{1} }} + \frac{hg}{\beta }} \right]\left( {\frac{{ - \left( {1 - e^{{ - \beta t_{1} }} } \right)}}{{t_{1}^{2} }} + \frac{{\beta e^{{ - \beta t_{1} }} }}{{t_{1} }}} \right) \hfill \\ \end{aligned} \right\} = 0$$

Again, the first term in the RHS of Eq. (21) is positive, so the second term equals to zero. In order to find the bounds for $t_{1}^{*}$, let

22 $$t_{{1{\text{U}}}}^{*} = \sqrt {\frac{{2\left[ {\beta \left( {K + nK_{1} } \right) + \delta_{4} } \right]}}{{P_{1} \beta \delta_{2} }}}$$

23 $$t_{{1{\text{L}}}}^{*} = {\text{the}}\;{\text{positive}}\;{\text{root}}\;{\text{of }}\left\{ {\frac{{ - \delta_{4} \pm \sqrt {\delta_{4}^{2} + 2P_{1} \left( {\delta_{2} + 2\beta \delta_{3} } \right)\left( {K + nK_{1} } \right)} }}{{P_{1} \left( {\delta_{2} + 2\beta \delta_{3} } \right)}}} \right\}$$

#### **Theorem 2**

$$t_{{1{\text{L}}}}^{*}$$ < $$t_{1}^{*}$$ < $$t_{{1{\text{U}}}}^{*}$$

To prove $t_{1}^{*}$ falls within bounds, we first multiply the second term of Eq. (21) by $\left( {2P_{1} t_{1}^{2} \beta } \right)$ and obtain

24 $$\left\{ {\left( {P_{1} \beta \delta_{2} + 2P_{1} \beta^{2} \delta_{3} e^{{ - \beta t_{1} }} } \right)t_{1}^{2} + \left( {2\delta_{4} \beta e^{{ - \beta t_{1} }} } \right)t_{1} - 2\left[ {\beta \left( {K + nK_{1} } \right) + \delta_{4} \left( {1 - e^{{ - \beta t_{1} }} } \right)} \right]} \right\} = 0$$

Thus

25 $$t_{1}^{*} = {\text{the}}\;{\text{positive}}\;{\text{root}}\;{\text{of}}\left\{ {\frac{{ - \left( {2\delta_{4} \beta e^{{ - \beta t_{1} }} } \right) \pm \sqrt {\left( {2\delta_{4} \beta e^{{ - \beta t_{1} }} } \right)^{2} - \left[ {4\left( {P_{1} \beta \delta_{2} + 2P_{1} \beta^{2} \delta_{3} e^{{ - \beta t_{1} }} } \right)\left[ { - 2\left[ {\beta \left( {K + nK_{1} } \right) + \delta_{4} \left( {1 - e^{{ - \beta t_{1} }} } \right)} \right]} \right]} \right]} }}{{2\left( {P_{1} \beta \delta_{2} + 2P_{1} \beta^{2} \delta_{3} e^{{ - \beta t_{1} }} } \right)}}} \right\}$$

In order to search for the optimal $t_{1}^{*}$, Eq. (24) can be rearranged as

26 $$2\left[ {P_{1} \beta^{2} \delta_{3} t_{1}^{2} + \delta_{4} \beta t_{1} + \delta_{4} } \right]\left( {e^{{ - \beta t_{1} }} } \right) = 2\left[ {\beta \left( {K + nK_{1} } \right) + \delta_{4} } \right] - \left( {P_{1} \beta \delta_{2} t_{1}^{2} } \right)$$

or

27 $$e^{{ - \beta t_{1} }} = \frac{{2\left[ {\beta \left( {K + nK_{1} } \right) + \delta_{4} } \right] - \left( {P_{1} \beta \delta_{2} t_{1}^{2} } \right)}}{{2\left[ {P_{1} \beta^{2} \delta_{3} t_{1}^{2} + \delta_{4} \beta t_{1} + \delta_{4} } \right]}}$$

Because $e^{{ - \beta t_{1} }}$ is the complement of the cumulative density function $F(t_{1} ) = 1 - e^{{ - \beta t_{1} }}$. As $0 \, \le \, F(t_{1} ) \, \le \, 1$, so $0 \, \le \, e^{{ - \beta t_{1} }} \le { 1}$. Let $e^{{ - \beta t_{1} }}$ = 0 and $e^{{ - \beta t_{1} }}$ = 1 represent bounds for $e^{{ - \beta t_{1} }}$, respectively. By substituting them into Eq. (25), we obtain

$$t_{{1{\text{U}}}}^{*} = \sqrt {\frac{{2\left[ {\beta \left( {K + nK_{1} } \right) + \delta_{4} } \right]}}{{P_{1} \beta \delta_{2} }}}$$

$$t_{{1{\text{L}}}}^{*} = {\text{the}}\;{\text{positive}}\;{\text{root}}\;{\text{of }}\;\left\{ {\frac{{ - \delta_{4} \pm \sqrt {\delta_{4}^{2} + 2P_{1} \left( {\delta_{2} + 2\beta \delta_{3} } \right)\left( {K + nK_{1} } \right)} }}{{P_{1} \left( {\delta_{2} + 2\beta \delta_{3} } \right)}}} \right\}$$

and $$t_{{1{\text{L}}}}^{*} < t_{1}^{*} < t_{{1{\text{U}}}}^{*}$$.

It can be seen that although the optimal $t_{1}^{*}$ cannot be expressed in a closed form, it falls within the aforementioned bounds, and it can be located with the use of a proposed recursive searching algorithm as follows.

Since $e^{{ - \beta t_{1} }}$ is the complement of cumulative density function, thus, $0 \, \le \, e^{{ - \beta t_{1} }} \le { 1}$.

Let

$$u(t_{1} ) = e^{{ - \beta t_{1} }} = \frac{{2\left[ {\beta \left( {K + nK_{1} } \right) + \delta_{4} } \right] - \left( {P_{1} \beta \delta_{2} t_{1}^{2} } \right)}}{{2\left[ {P_{1} \beta^{2} \delta_{3} t_{1}^{2} + \delta_{4} \beta t_{1} + \delta_{4} } \right]}}\quad \therefore 0 \le u(t_{1} ) \le 1$$

The proposed recursive searching algorithm to find $t_{1}^{*}$ is listed below:

1. Let *u*(*t*_1_) = 0 and *u*(*t*_1_) = 1 initially and compute the upper and lower bounds for $t_{1}^{*}$, respectively (i.e., to obtain the initial values of [$t_{{1{\text{L}}}}^{*}$, $t_{{1{\text{U}}}}^{*}$]).
2. Substitute the current values of [$t_{{1{\text{L}}}}^{*}$, $t_{{1{\text{U}}}}^{*}$] into $e^{{ - \beta t_{1} }}$ and calculate the new bounds (expressed as *u*_L_ and *u*_U_) for $e^{{ - \beta t_{1} }}$. Hence, *u*_L_ < *u*(*t*_1_) < *u*_U_.
3. Let *u*(*t*_1_) = *u*_L_ and *u*(*t*_1_) = *u*_U_, and re-compute the new upper and lower bounds for $t_{1}^{*}$, respectively (i.e. to update the current values of [$t_{{1{\text{L}}}}^{*}$, $t_{{1{\text{U}}}}^{*}$]).
4. Repeat steps 2 and 3, until there is no significant difference between $t_{{1{\text{L}}}}^{*}$ and $t_{{1{\text{U}}}}^{*}$ (or there is no significant difference in terms of their effects on E[*TCU*($t_{1}^{*}$)]).
5. Stop. The optimal production run time $t_{1}^{*}$ is obtained.

## Model 2: Extension to a producer–retailer integrated system

### Problem description and modeling

**Fig. 4:**
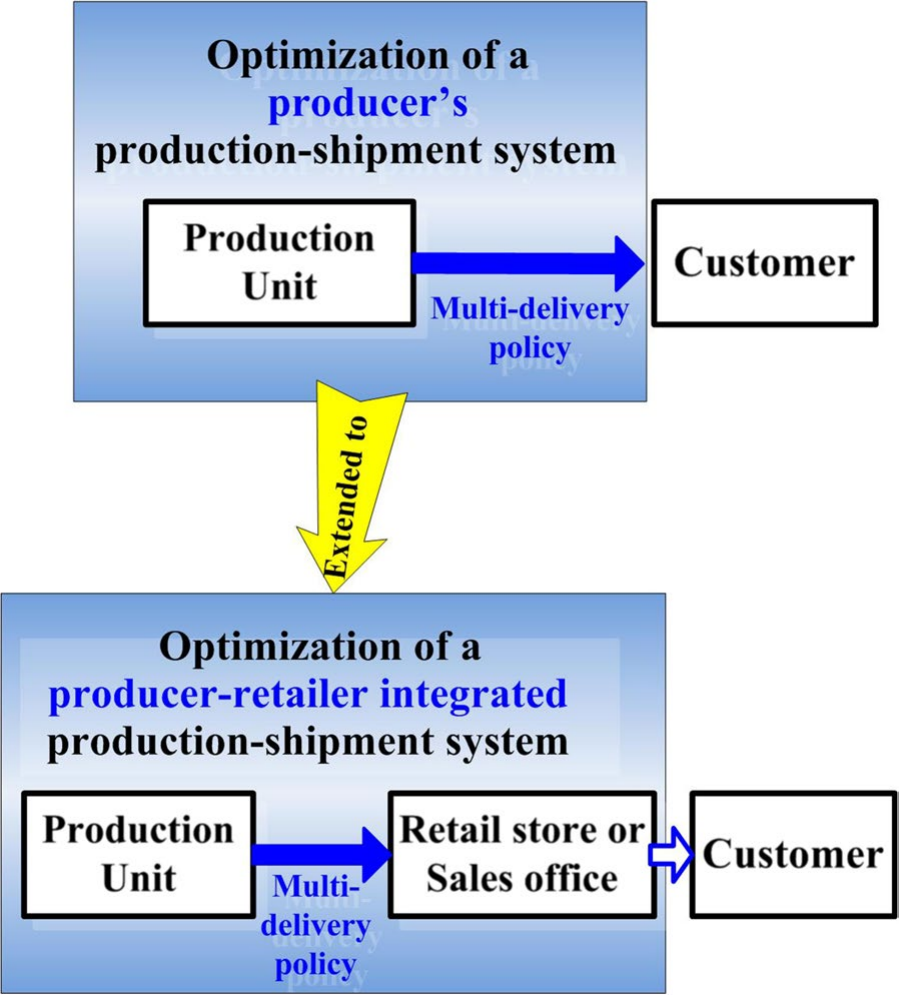
Extension to a producer-retailer integrated EMQ system (model 2)

In the manufacturing sector, some producers of consumer goods may have their own retail stores or regional sales offices to promote and sell their finished products to customers (Fig. 4). In order to address such a real life intra-supply chains situation, the second model of this study enhances the first model to further explore the optimal run time for a producer–retailer integrated EMQ model, wherein, the retailer’s stock holding cost is incorporated into the first model.

**Fig. 5:**
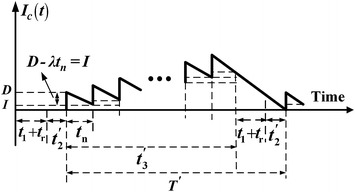
On-hand Inventory level of finished products at the retailer’s side in the extended EMQ model 2 with breakdown occurrence

According to the proposed multi-delivery policy, Fig. 5 depicts the stock holding status at the retailer’s side for the case of breakdown taking place. Additional notations used at the modeling and analysis of this extended producer–retailer integrated EMQ model 2 are listed as follows.

*D*: number of finished items (a fixed quantity) distributed to retail store per delivery,
*I*: number of left over items in each interval of time *t*_n_, after satisfying the demand in *t*_n_,
*I*_c_(*t*): on-hand inventory level of finished products at the retailer’s side at time *t*,
*h*_2_: unit holding cost for products stored at the retailer’s side,
*TC*_3_(*t*_1_): total production–inventory–delivery costs per cycle in this enhanced model (in the case of breakdown occurrence),
*TC*_4_(*t*_1_): total production–inventory–delivery costs per cycle in this enhanced model (in the case of no breakdown occurrence),
*E*[*TC*_3_(*t*_1_)]: the expected production–inventory–delivery costs per cycle in this enhanced model (in the case of breakdown occurrence),
*E*[*TC*_4_(*t*_1_)]: the expected production–inventory–delivery costs per cycle in this enhanced model (in the case of no breakdown occurrence),
*E*[*TCU*_2_(*t*_1_)]: the long-run expected production–inventory–delivery costs per unit time in this enhanced model, whether a breakdown occurring or not.

It can be seen from Fig. 5, at the retailer’s side the demand between shipments is *λt*_n_, the number of left over items in each interval of time *t*_n_ (after satisfying the demand in *t*_n_) is

28 $$I = D - \lambda \, t_{n}$$

Total inventory holding costs on the retailer’s side with and without breakdown are expressed in Eqs. (29) and (30), respectively (also refer to Fig. 5):

29 $$h_{2} \left[ {n\frac{{\left( {D - I} \right)}}{2}t_{n} + \frac{n(n + 1)}{2}It_{n} + \frac{nI}{2}\left( {t_{1} + t_{r} + t_{2}^{'} } \right)} \right]$$

30 $$h_{2} \left[ {n\frac{{\left( {D - I} \right)}}{2}t_{n} + \frac{n(n + 1)}{2}It_{n} + \frac{nI}{2}\left( {t_{1} + t_{2} } \right)} \right]$$

By incorporating the retailer’s holding costs into the original models with and without breakdown, respectively (i.e., into Eqs. (4) and (6) in “Model 1: Description, modeling, and solution process” section), we obtain

31 $$TC_{3} \left( {t_{1} } \right) = TC_{1} \left( {t_{1} } \right) + h_{2} \left[ {n\frac{{\left( {D - I} \right)}}{2}t_{n} + \frac{n(n + 1)}{2}It_{n} + \frac{nI}{2}\left( {t_{1} + t_{r} + t_{2}^{'} } \right)} \right]$$

32 $$TC_{4} \left( {t_{1} } \right) = TC_{2} \left( {t_{1} } \right) + h_{2} \left[ {n\frac{{\left( {D - I} \right)}}{2}t_{n} + \frac{n(n + 1)}{2}It_{n} + \frac{nI}{2}\left( {t_{1} + t_{2} } \right)} \right]$$

To take the randomness of defective items into account and substitute all related parameters into Eqs. (31) and (32), and with further derivations, we obtain *E*[*TC*_3_(*t*_1_)] and *E*[*TC*_4_(*t*_1_)] as

33 $$E\left[ {TC_{3} \left( {t_{1} } \right)} \right] = E\left[ {TC_{1} \left( {t_{1} } \right)} \right] + h_{2} \left( {\frac{{P_{1} g\left( {1 - \theta_{1} E[x]} \right)}}{2}} \right)\left( {1 - \frac{1}{n}} \right)t_{1} + h_{2} \left( {\frac{{P_{1} \left( {1 - \theta_{1} E[x]} \right)}}{2}} \right)\left[ \begin{aligned} \frac{{P_{1} \left( {1 - \theta_{1} E[x]} \right)}}{\lambda n} \hfill \\ + \left( {1 - \frac{1}{n}} \right)\left( {1 + \frac{{P_{1} E[x]}}{{P_{2} }}} \right) \hfill \\ \end{aligned} \right]t_{1}^{2}$$

34 $$E\left[ {TC_{4} \left( {t_{1} } \right)} \right] = E\left[ {TC_{2} \left( {t_{1} } \right)} \right] + h_{2} \left( {\frac{{P_{1} \left( {1 - \theta_{1} E[x]} \right)}}{2}} \right)\left[ \begin{aligned} \frac{{P_{1} \left( {1 - \theta_{1} E[x]} \right)}}{\lambda n} \hfill \\ + \left( {1 - \frac{1}{n}} \right)\left( {1 + \frac{{P_{1} E[x]}}{{P_{2} }}} \right) \hfill \\ \end{aligned} \right]t_{1}^{2}$$

### Integration of producer–retailer integrated models with and without breakdown

As stated in section “Integration of the proposed EMQ models with and without breakdown”, in the proposed study a breakdown can occur randomly and the mean time to machine breakdowns obeys an exponential distribution with density function $f\left( t \right) = \beta e^{ - \beta t}$, and its cumulative density function ${\text{F}}\left( t \right) = 1 - e^{ - \beta t}$. Hence, the expected production–inventory–delivery costs per unit time, E[*TCU*_2_(*t*_1_)] is

35 $$E\left[ {TCU_{2} \left( {t_{1} } \right)} \right] = \frac{{\left\{ {\int_{ \, 0}^{{ \, t_{1} }} {E\left[ {TC_{3} \left( {t_{1} } \right)} \right]} f\left( t \right)dt + \int_{{ \, t_{1} }}^{ \, \infty } {E\left[ {TC_{4} \left( {t_{1} } \right)} \right]} f\left( t \right)dt} \right\}}}{{{\text{E}}[\varvec{T}]}}$$

By substituting E[*TC*_3_(*t*_1_)], E[*TC*_4_(*t*_1_)], and E[***T***] into Eq. (35) and solving the integration of the mean time to breakdown in E[*TCU*_2_(*t*_1_)], we obtain

36 $$\begin{aligned} E\left[ {TCU_{2} (t_{1} )} \right] = \frac{\lambda }{{\left( {1 - \theta_{1} E[x]} \right)}} \cdot \hfill \\ \quad \quad \left\{ \begin{aligned} &\frac{{\left( {K + nK_{1} } \right)}}{{t_{1} P_{1} }} + \delta_{1} + \frac{{\delta_{2} t_{1} }}{2} + \delta_{5} t_{1} + \left[ {\frac{M}{{P_{1} }} + \frac{hg}{\beta }} \right]\left( {\frac{{1 - e^{{ - \beta t_{1} }} }}{{t_{1} }}} \right) \hfill \\ &- hg\left( {e^{{ - \beta t_{1} }} } \right) - \left( {h - h_{2} } \right)\left[ {\frac{{g\left( {1 - \theta_{1} E[x]} \right)}}{2}\left( {1 - \frac{1}{n}} \right)} \right]\left( {1 - e^{{ - \beta t_{1} }} } \right) \hfill \\ \end{aligned} \right\} \hfill \\ \end{aligned}$$

where *δ*_1_ and *δ*_2_ are provided in Eqs. (11) and (12), and let *δ*_5 is_ denote the following

37 $$\delta_{5} = \frac{{h_{2} \left( {1 - \theta_{1} E[x]} \right)}}{2}\left[ {\frac{{P_{1} \left( {1 - \theta_{1} E[x]} \right)}}{\lambda n} + \left( {1 - \frac{1}{n}} \right)\left( {1 + \frac{{P_{1} E[x]}}{{P_{2} }}} \right)} \right]$$

### Determining the optimal production run time

Before determining the optimal production run time $t_{1}^{*}$, we must first prove that E[*TCU*_2_(*t*_1_)] is convex. Let *π*(*t*_1_) represent the following:

38 $$\pi \left( {t_{1} } \right) = \frac{{2\left( {K + nK_{1} } \right)\beta + 2\left( {1 - e^{{ - \beta t_{1} }} } \right)\delta_{4} }}{{\left[ {t_{1}^{2} P_{1} \beta^{2} \delta_{6} + \delta_{4} \left( {2 + \beta t_{1} } \right)} \right]\beta e^{{ - \beta t_{1} }} }}$$

#### **Theorem 3**

*E*[*TCU*_2_(*t*_1_)] *is convex if 0* < *t*_1_ < *π*(*t*_1_).

Equation (39) shows the second derivative of *E*[*TCU*_2_(*t*_1_)] with respect to *t*_1_.

39 $$\frac{{d^{2} E[TCU_{2} (t_{1} )]}}{{d^{2} t_{1}^{2} }} = \frac{\lambda }{{\left( {1 - \theta_{1} E[x]} \right)}}\left[ \begin{aligned} &\frac{{2\left( {K + nK_{1} } \right)}}{{t_{1}^{3} P_{1} }} + \left( {h - h_{2} } \right)\frac{{g\left( {1 - \theta_{1} E[x]} \right)}}{2}\left( {1 - \frac{1}{n}} \right)\left( {\beta^{2} e^{{ - \beta t_{1} }} } \right) \hfill \\ &- hg\left( {\beta^{2} e^{{ - \beta t_{1} }} } \right) + \left[ {\frac{M}{{P_{1} }} + \frac{hg}{\beta }} \right]\left( {\frac{{2\left( {1 - e^{{ - \beta t_{1} }} } \right)}}{{t_{1}^{3} }} - \frac{{2\beta e^{{ - \beta t_{1} }} }}{{t_{1}^{2} }} - \frac{{\beta^{2} e^{{ - \beta t_{1} }} }}{{t_{1} }}} \right) \hfill \\ \end{aligned} \right]$$

The first term in the RHS of Eq. (39) is positive because annual demand *λ* > 0. Therefore, we have

40 $${\text{if}}\left[ \begin{aligned} \frac{{2\left( {K + nK_{1} } \right)}}{{t_{1}^{3} P_{1} }} + \left( {h - h_{2} } \right)\left[ {\frac{{g\left( {1 - \theta_{1} E[x]} \right)}}{2}\left( {1 - \frac{1}{n}} \right)} \right]\left( {\beta^{2} e^{{ - \beta t_{1} }} } \right) \hfill \\ - hg\left( {\beta^{2} e^{{ - \beta t_{1} }} } \right) + \left[ {\frac{M}{{P_{1} }} + \frac{hg}{\beta }} \right]\left( {\frac{{2\left( {1 - e^{{ - \beta t_{1} }} } \right)}}{{t_{1}^{3} }} - \frac{{2\beta e^{{ - \beta t_{1} }} }}{{t_{1}^{2} }} - \frac{{\beta^{2} e^{{ - \beta t_{1} }} }}{{t_{1} }}} \right) \hfill \\ \end{aligned} \right] > 0\quad {\text{then}}\quad \frac{{d^{2} E\left[ {TCU_{2} \left( {t_{1} } \right)} \right]}}{{dt_{1}^{2} }} > 0$$

With further derivations, the left-hand side (LHS) of Eq. (40) becomes

41 $${\text{if}}\left[ \begin{aligned} &2\left( {K + nK_{1} } \right)\beta - t_{1}^{3} P_{1} \beta \left( {h - h_{2} } \right)\left[ {\frac{{g\left( {1 - \theta_{1} E[x]} \right)}}{2}\left( {1 - \frac{1}{n}} \right)} \right]\left( {\beta^{2} e^{{ - \beta t_{1} }} } \right) \hfill \\ &- t_{1}^{3} P_{1} \beta hg\left( {\beta^{2} e^{{ - \beta t_{1} }} } \right) + \left( {M\beta + hgP_{1} } \right)\left[ {2\left( {1 - e^{{ - \beta t_{1} }} } \right) - 2t_{1} \beta e^{{ - \beta t_{1} }} - \beta^{2} t_{1}^{2} e^{{ - \beta t_{1} }} } \right] \hfill \\ \end{aligned} \right] > 0 \,$$

Let

$$\delta_{6} = \left\{ {\left( {h - h_{2} } \right)\left[ {\frac{{g\left( {1 - \theta_{1} E[x]} \right)}}{2}\left( {1 - \frac{1}{n}} \right)} \right] + hg} \right\}\quad {\text{and}}\;{\text{recall}}\;\delta_{4} = \left( {M\beta + hgP_{1} } \right),$$

Then Eq. (40) becomes

42 $${\text{if}}\left[ \begin{aligned} &2\left( {K + nK_{1} } \right)\beta + 2\left( {1 - e^{{ - \beta t_{1} }} } \right)\delta_{4} \hfill \\& - t_{1} \left[ {t_{1}^{2} P_{1} \beta^{2} \delta_{6} + \delta_{4} \left( {2 + \beta t_{1} } \right)} \right]\beta e^{{ - \beta t_{1} }} \hfill \\ \end{aligned} \right] > 0\quad {\text{then}}\;\frac{{d^{2} E\left[ {TCU_{2} \left( {t_{1} } \right)} \right]}}{{dt_{1}^{2} }} > 0$$

or

43 $$\frac{{d^{2} \, E\left[ {TCU_{2} \left( {t_{1} } \right)} \right]}}{{dt_{1}^{2} }} > 0\quad {\text{if}}\quad 0 < t_{1} < \frac{{2\left( {K + nK_{1} } \right)\beta + 2\left( {1 - e^{{ - \beta t_{1} }} } \right)\delta_{4} }}{{\left[ {t_{1}^{2} P_{1} \beta^{2} \delta_{6} + \delta_{4} \left( {2 + \beta t_{1} } \right)} \right]\beta e^{{ - \beta t_{1} }} }} = \pi \left( {t_{1} } \right)$$

If *E*[*TCU*_2_(*t*_1_)] is proven to be convex, then the optimal production run time $t_{1}^{*}$ can be solved by setting the first derivative of E[*TCU*_2_ (*t*_1_)] = 0.

44 $$\frac{{dE[TCU_{2} (t_{1} )]}}{{dt_{1} }} = \frac{\lambda }{{\left( {1 - \theta_{1} E[x]} \right)}} \cdot \left\{ \begin{aligned} \frac{{ - \left( {K + nK_{1} } \right)}}{{t_{1}^{2} P}} + \left( {\frac{{\delta_{2} }}{2} + \delta_{5} } \right) + \delta_{6} \left( {\beta e^{{ - \beta t_{1} }} } \right) \hfill \\ + \left[ {\frac{M}{{P_{1} }} + \frac{hg}{\beta }} \right]\left( {\frac{{ - \left( {1 - e^{{ - \beta t_{1} }} } \right)}}{{t_{1}^{2} }} + \frac{{\beta e^{{ - \beta t_{1} }} }}{{t_{1} }}} \right) \hfill \\ \end{aligned} \right\} = 0$$

Since the first term in the RHS of Eq. (44) is positive, and so the second term is equal to zero. In order to find the bounds for $t_{1}^{*}$, let

45 $$t_{{1{\text{U}}}}^{*} = \sqrt {\frac{{2\left[ {\beta \left( {K + nK_{1} } \right) + \delta_{4} } \right]}}{{P_{1} \beta \left( {\delta_{2} + 2\delta_{5} } \right)}}}$$

46 $$t_{{1{\text{L}}}}^{*} = {\text{the}}\;{\text{positive}}\;{\text{root}}\;{\text{of}}\;\left\{ {\frac{{ - \delta_{4} \pm \sqrt {\delta_{4}^{2} + 2P_{1} \left( {K + nK_{1} } \right)\left( {\delta_{2} + 2\delta_{5} + 2\beta \delta_{6} } \right)} }}{{P_{1} \left( {\delta_{2} + 2\delta_{5} + 2\beta \delta_{6} } \right)}}} \right\}$$

#### **Theorem 4**

$$t_{{1{\text{L}}}}^{*}$$ < $$t_{1}^{*}$$ < $$t_{{1{\text{U}}}}^{*}$$

Please refer to the similar proof as that for Theorem 2 (presented in “Model 1: Description, modeling, and solution process” section).

Upon showing that $t_{1}^{*}$ falls within the upper and lower bounds, by multiplying the second term of Eq. (44) by $\left( {2P_{1} t_{1}^{2} \beta } \right)$ one has

47 $$\left\{ {\left[ {P_{1} \beta \left( {\delta_{2} + 2\delta_{5} } \right) + 2P_{1} \beta^{2} \delta_{6} e^{{ - \beta t_{1} }} } \right]t_{1}^{2} + \left( {2\delta_{4} \beta e^{{ - \beta t_{1} }} } \right)t_{1} - 2\left[ {\beta \left( {K + nK_{1} } \right) + \delta_{4} \left( {1 - e^{{ - \beta t_{1} }} } \right)} \right]} \right\} = 0$$

With further rearrangement, one obtains

48 $$e^{{ - \beta t_{1} }} = \frac{{2\left[ {\beta \left( {K + nK_{1} } \right) + \delta_{4} } \right] - \left[ {P_{1} \beta \left( {\delta_{2} + 2\delta_{5} } \right)} \right]t_{1}^{2} }}{{2\left[ {P_{1} \beta^{2} \delta_{6} t_{1}^{2} + \delta_{4} \beta t_{1} + \delta_{4} } \right]}}$$

Because $e^{{ - \beta t_{1} }}$ is the complement of the cumulative density function $F(t_{1} ) = 1 - e^{{ - \beta t_{1} }}$. As $0 \, \le \, F(t_{1} ) \, \le \, 1$, so $0 \, \le \, e^{{ - \beta t_{1} }} \le { 1}$. Let $e^{{ - \beta t_{1} }}$ = 0 and $e^{{ - \beta t_{1} }}$ = 1 represent the upper and lower bounds of $e^{{ - \beta t_{1} }}$, respectively. Then, by applying the proposed recursive searching algorithm provided at the end of section “Model 1: Description, modeling, and solution process”, we can find the optimal production run time $t_{1}^{*}$.

## Numerical Examples

### Numerical demonstration of proposed EMQ model 1

This section uses the same numerical example as in Chiu et al. (2009) to provide a comparison for readers. The following values are used for the corresponding system variables:

*β*: Poisson breakdown rate with a mean of 0.5 per year,
*M*: fixed machine repair cost of $500 per breakdown,
*g*: machine repair time (*t*_r_) of 0.018 year,
*λ*: demand rate of 4000 items per year,
*P*_1_: production rate of 10,000 items per year,
*x*: uniform distribution nonconforming rate over the range [0, 0.2],
*K*: setup cost of $450 per cycle,
*C*: unit manufacturing cost of $2,
*h*: holding cost of $0.6 per item per unit time,
*P*_2_: rework rate of 5000 items per year,
*C*_R_: unit rework cost of $0.50 per product per year,
*h*_1_: holding cost of $0.80 for each reworked item,
*θ*_1_: rework failure rate of 0.1,
*C*_S_: unit disposal cost of $0.3 for scrap items,
*K*_1_: fixed transportation cost of $90 per delivery,
*n*: number of deliveries per cycle: 4,
*C*_T_: variable delivery cost of $0.001 per product shipped

To test for the convexity of E[*TCU(t*_1_*)*] (Theorem 1), we can apply the upper and lower bounds of $t_{1}^{*}$ (i.e., Eqs. (22) and (23)) to Eq. (13), and obtain that $t_{{1{\text{U}}}}^{*}$ = 0.4595 < *w*($t_{{1{\text{U}}}}^{*}$) = 2.87230, and $t_{{1{\text{L}}}}^{*}$ = 0.3056 < *w*($t_{{1{\text{L}}}}^{*}$) = 2.62608. Therefore, the expected costs function [*TCU*(*t*_1_)] is convex.

**Fig. 6:**
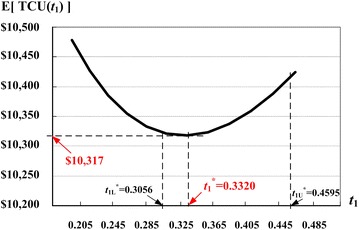
The effects of variations in the production run time *t*_1_ on E[*TCU*(*t*_1_)] in the extended EMQ model 1

Next, to find the optimal production run time $$t_{1}^{*}$$, we substitute $$t_{{1{\text{U}}}}^{*}$$ and $$t_{{1{\text{L}}}}^{*}$$ into Eq. (10), and find that E[*TCU*($$t_{{1{\text{U}}}}^{*}$$)] = $10,417.21 and E[*TCU*($$t_{{1{\text{L}}}}^{*}$$)] = $10,323.69, respectively. Since the optimal run time $$t_{1}^{*}$$ falls within the bounds of $$t_{{1{\text{L}}}}^{*}$$ and $$t_{{1{\text{U}}}}^{*}$$, by applying the proposed searching algorithm, we find that $$t_{1}^{*}$$ = 0.3320 years and that the optimal system cost E[*TCU*($$t_{1}^{*}$$)] = $10,317.27. Figure 6 depicts the effect of variations in production run time *t*_1_ on the expected system cost E[*TCU*(*t*_1_)]. Table 1 exhibits the step-by-step iterations of the proposed searching algorithm.

**Table 1:** Iterations of the proposed searching algorithm for $t_{1}^{*}$ for the extended EMQ model 1

*β*	Step #	$$t_{{1{\text{U}}}}^{*}$$	*u*_L_ = $$e^{{ - \beta t_{{1{\text{U}}}} }}$$	$$t_{{1{\text{L}}}}^{*}$$	*u*_U_ = $$e^{{ - \beta t_{{1{\text{L}}}} }}$$	Difference between $$t_{{1{\text{U}}}}^{*}$$ and $$t_{{1{\text{L}}}}^{*}$$	[U]E[*TCU*($$t_{{1{\text{U}}}}^{*}$$)]	[L]E[*TCU*($$t_{{1{\text{L}}}}^{*}$$)]	Difference between [U] and [L]
0.5	Initial	–	0.0000	–	1.0000	–	–	–	–
	1st	0.4595	0.7947	0.3056	0.8583	0.1539	$10,417.21	$10,323.69	$93.52
	2nd	0.3407	0.8434	0.3301	0.8479	0.0106	$10,317.90	$10,317.30	$0.60
	3rd	0.3326	0.8468	0.3318	0.8472	0.0008	$10,317.28	$10,317.27	$0.01
	4th	0.3321	0.8470	0.3320	0.8471	0.0001	$10,317.27	$10,317.27	$0.00
	5th	0.3320	0.8471	0.3320	0.8471	0.0000	$10,317.27	$10,317.27	$0.00

The proposed EMQ-based model 1 is intended to address the effect of discontinuous multi-delivery policy on the EMQ model with machine breakdown and rework failures. For this reason, sensitivity analyses of variations in the fixed transportation cost and their effects on the expected system cost E[*TCU*($t_{1}^{*}$)] and on the production run time $t_{1}^{*}$ are carried out.

**Fig. 7:**
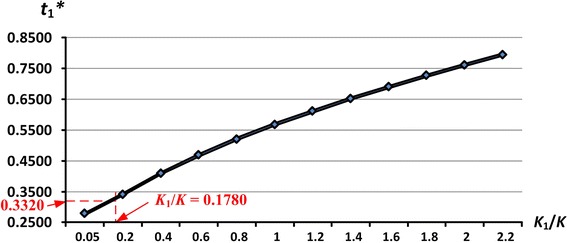
The effects of variations in the *K*_1_/*K* ratios on the optimal $t_{1}^{*}$ in the extended EMQ model 1

**Fig. 8:**
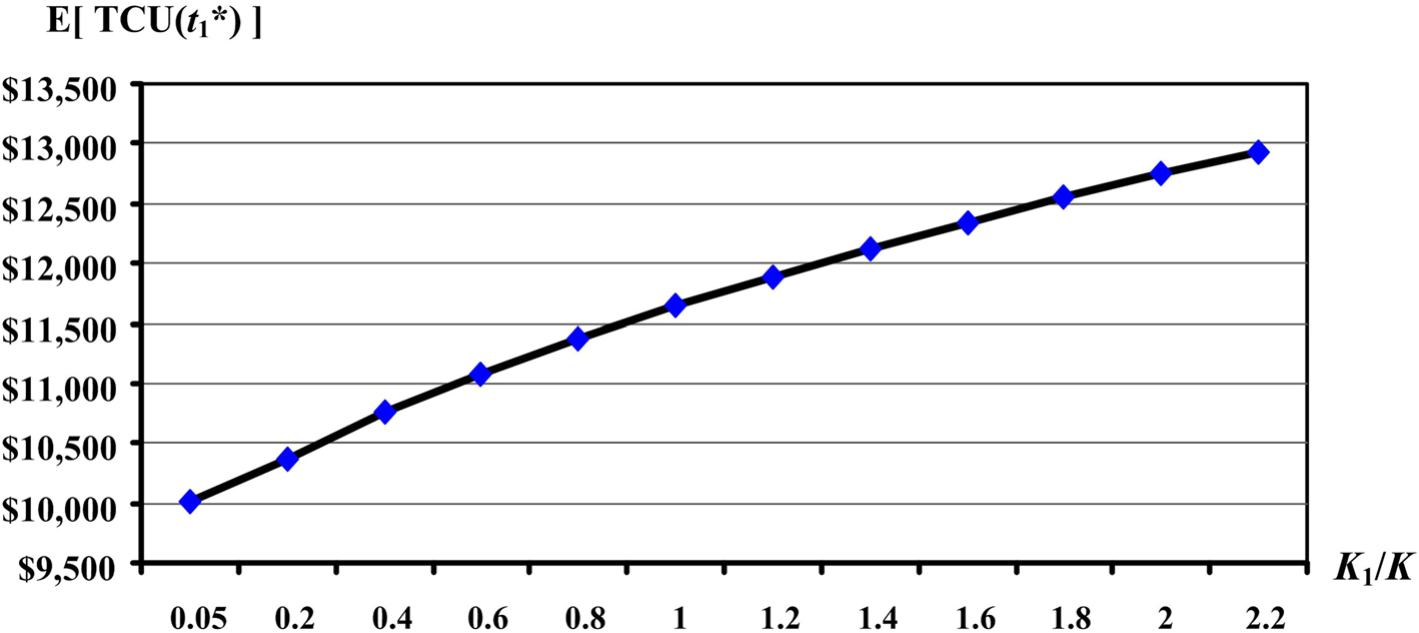
The effects of variations in the *K*_1_/*K* ratios on the optimal E[*TCU*($t_{1}^{*}$)] in the extended EMQ model 1

The results indicate that, as the fixed transportation costs *K*_1_ (or the ratio of *K*_1_/*K*) increase, the production run time $t_{1}^{*}$ increases significantly (as illustrated in Fig. 7) along with the expected system cost E[*TCU*($t_{1}^{*}$)] (see Fig. 8).

### Numerical demonstration of proposed EMQ model 2

This subsection provides a numerical example to explain the proposed producer–retailer integrated EMQ model 2. First, we assume an extra parameter-unit holding cost on the retailer’s side *h*_2_ = $1.50. By applying Eqs. (45) and (46), we obtain that $$t_{{1{\text{U}}}}^{*}$$ = 0.2848 and $$t_{{1{\text{L}}}}^{*}$$ = 0.1951, respectively. Then, we use them to test for the convexity of the system cost E[*TCU*($$t_{1}^{*}$$)] (Theorem 3) and find that $$t_{{1{\text{U}}}}^{*}$$ < *π*($$t_{{1{\text{U}}}}^{*}$$) = 2.5894 and $$t_{{1{\text{L}}}}^{*}$$ < *π*($$t_{{1{\text{L}}}}^{*}$$) = 2.4511. Hence, the system cost function [*TCU*(*t*_1_)] is convex.

Once the convexity of [*TCU*(*t*_1_)] is proven, we apply the proposed recursive algorithm (Theorem 4) to search for the optimal run time $$t_{1}^{*}$$ over the interval [$$t_{{1{\text{L}}}}^{*}$$, $$t_{{1{\text{U}}}}^{*}$$]. By substituting $$t_{{1{\text{U}}}}^{*}$$ and $$t_{{1{\text{L}}}}^{*}$$ in Eq. (36), we obtain the initial values of E[*TCU*($$t_{{1{\text{U}}}}^{*}$$)] = $11,642.46 and E[*TCU*($$t_{{1{\text{L}}}}^{*}$$)] = $11,481.07. Then, by further applying the algorithm (as provided at the end of “Model 1: Description, modeling, and solution process” section), we find that $$t_{1}^{*}$$ = 0.2051 years and E[*TCU*($$t_{1}^{*}$$)] = $11,477.27. The step-by-step iterative results are shown in Table 2.

**Table 2:** Iterations of the proposed searching algorithm for $t_{1}^{*}$ for the extended EMQ model 2

*β*	Step #	$$t_{{1{\text{U}}}}^{*}$$	*u*_L_ = $$e^{{ - \beta t_{{1{\text{U}}}} }}$$	$$t_{{1{\text{L}}}}^{*}$$	*u*_U_ = $$e^{{ - \beta t_{{1{\text{L}}}} }}$$	Difference between $$t_{{1{\text{U}}}}^{*}$$ and $$t_{{1{\text{L}}}}^{*}$$	[U] E[*TCU*($$t_{{1{\text{U}}}}^{*}$$)]	[L] E[*TCU*($$t_{{1{\text{L}}}}^{*}$$)]	Difference between [U] and [L]
0.5	Initial	–	0.0000	–	1.0000	–	–	–	–
	1st	0.2848	0.8673	0.1951	0.9071	0.0898	$11,642.46	$11,481.07	$161.39
	2nd	0.2086	0.9009	0.2046	0.9027	0.0040	$11,477.72	$11,477.28	$0.44
	3rd	0.2052	0.9025	0.2051	0.9026	0.0001	$11,477.28	$11,477.27	$0.01
	4th	0.2051	0.9025	0.2051	0.9025	0.0000	$11,477.27	$11,477.27	$0.00

**Fig. 9:**
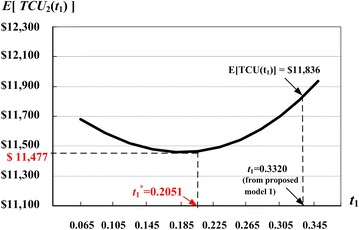
The behavior of E[*TCU*_2_(*t*_1_)] and *t*_1_ in the extended EMQ model 2

A further analysis on the behavior of the system costs E[*TCU*(*t*_1_)] related to the production run time *t*_1_ is depicted in Fig. 9. It shows that, by simply applying the result derived from the second EMQ model, the management of such a producer–retailer integrated system can help realize cost savings of $359.00 (or 3.13 % over the total system costs) as compared to the result from the EMQ model 1.

**Fig. 10:**
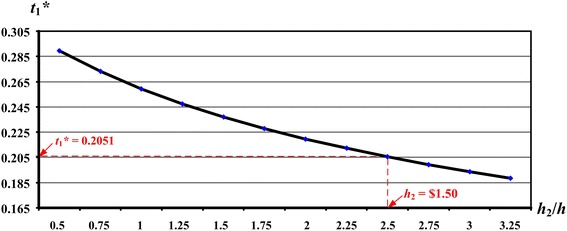
The effects of variations in the *h*_2_/*h* ratios on the optimal $t_{1}^{*}$ in the extended EMQ model 2

**Fig. 11:**
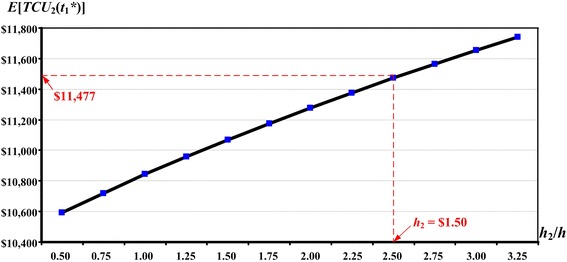
The effects of variations in the *h*_2_/*h* ratios on the optimal E[*TCU*_2_($t_{1}^{*}$)] in the extended EMQ model 2

The purpose of the proposed EMQ-based model 2 is to explore the optimal run time $t_{1}^{*}$ for a producer–retailer integrated EMQ model and to study the effect of the retailer’s holding costs on the replenishment decision. For this reason, sensitivity analyses of variations in *h*_2_ (or the ratio of *h*_2_/*h*) and their effects on $t_{1}^{*}$ and the expected system cost E[*TCU*($t_{1}^{*}$)] are performed (see Figs. 10, 11).

The figures show that, as the retailer’s holding costs *h*_2_ (or the ratio of *h*_2_/*h*) increase, the optimal production run time $t_{1}^{*}$ decreases and the expected cost E[*TCU*($t_{1}^{*}$)] increases. For management of the producer–retailer integrated system, the results of these sensitivity analyses can provide helpful information during the decision-making process, by offering insights into the effects of various inventory-holding costs in different retail stores.

## Conclusions

In this study, two extended EMQ-based models with a discontinuous product issuing policy, random machine breakdown, and rework failures are developed. Various real conditions in the production process, end-product delivery, and intra-supply chains such as a producer–retailer integrated scheme are examined. With the aid of mathematical modeling and optimization techniques, we derive the optimal replenishment run time decision and reveal various important factors for the system parameters of the studied models (refer to Figs. 6, 7, 8, 9, 10, 11 as examples).

The obtained results can help production planners determine the optimal production run time in an intra-supply chain situations where finished products are distributed to companies’ own retail stores or regional sales offices and stocked there for sale. An interesting area for future study would be the effect of variable production rates on these models.
